# Effects of Different Lignins on Methane Separation from Coalbed Methane Gas via Hydrate Formation Under Pressure Stabilization and Pressure Variation Systems

**DOI:** 10.3390/ma19214737

**Published:** 2026-11-07

**Authors:** Kun Ding, Xuetao Wang, Xiaokun Miao

**Affiliations:** 1 College of Physics and New Energy, Xuzhou University of Technology, Xuzhou 221018, China; 2 International Joint Research Laboratory for Energy Power Equipment Energy Conservation and Pollutant Control of Henan, Henan University of Science and Technology, Luoyang 471003, China

**Keywords:** pressure stabilization system, gas uptake, methane recovery, lignin, hydrate formation

## Abstract

In this study, a new pressure stabilization (PS) system composed of a gas-stable vessel (SV), a PID control system, and a reaction vessel (RV) was used to investigate the dynamic characteristics of methane separation from low-concentration coalbed methane and to compare its performance with that of a pressure variation (PV) system. A bio-surfactant sulfonated lignin system was constructed using sodium lignosulfonate (SL) or calcium lignosulfonate (CL) solution, which reduced gas–liquid interfacial tension and enhanced methane dissolution and mass transfer. In addition, cyclopentane (CP) was introduced to the solution as a thermodynamic accelerator to reduce the hydration reaction conditions and improve its methane storage capacity. The experiments were carried out at 275.15 K and 3.0 MPa; the mass concentration of the lignin was 500 ppm and the volume ratio of CP to deionized water was 1:10. The results showed that the PS + SL + CP system achieved the highest CH_4_ recovery (66.5%) and gas uptake (0.1142 mol), whereas the PV + SL + CP system exhibited a shorter *t*_90_ (156.7 min) and a higher CH_4_ concentration (69.5%) in the hydrate phase. In contrast, the shortest induction time (10.3 min) was observed for the PS + CL + CP system.

## 1. Introduction

Coalbed methane, an unconventional natural gas resource with abundant reserves and clean-burning properties, is an important supplement to the global natural gas supply and is typically found at depths of up to 2000 m [1,2]. Low-concentration coalbed methane (LCM) contains a high proportion of nitrogen, along with trace amounts of carbon dioxide and oxygen; it is extracted from underground coal mines, with a methane volume fraction generally below 30% [3]. The direct pipeline transportation and large-scale utilization of LCM remain difficult. As a result, large quantities of gas are usually vented on site, resulting in a significant waste of clean energy, exacerbating greenhouse gas emissions, and increasing the risk of mining gas disasters [4]. The development of economical, mild, and environmentally friendly technologies for the enrichment and separation of LCM [5,6] has important theoretical and engineering value for the low-carbon transformation of the coal industry and the improvement of resource utilization efficiency.

However, conventional separation processes, such as pressure swing adsorption, cryogenic liquefaction, and membrane separation, have substantial drawbacks. For example, PSA generally provides limited methane separation efficiency [7], whereas membrane separation is often associated with high operational costs [6]. Gas hydrate crystallization, whereby water molecules rely on hydrogen bonds to build cage-shaped lattices that encapsulate gas guest molecules such as CH_4_ and CO_2_, forming cage-shaped hydrates, has been considered as a promising method for the separation of gas mixtures due to its high gas storage capacity and moderate temperature/pressure formation conditions [8,9,10]. However, methane separation from LCM via hydrate formation has mainly been studied under pressure variation (PV) conditions, where the pressure in the reactor gradually decreases as the hydration reaction progresses. For example, the pressure drop (Δ*P*) of the reactor can reach 0.4 MPa [10], and even 1.2 MPa [9], which would reduce the CH_4_ content in the gas phase of the reactor and gradually decrease the rate of hydration reaction. The new pressure stabilization (PS) system based on the existing PV experimental system [10,11] was designed for experimental research on hydrate formation. The pressure in the gas phase of the reactor under a PS system remains relatively constant as a result of the continuous inflow of precooled fresh LCM, thus ensuring the hydration reaction proceeds under relatively stable pressure conditions. This represents the innovation of this work.

Previous studies have reported that high methane storage capacities can be achieved from LCM by constructing a bio-surfactant sulfonated lignin system through the addition of carbon, lignin [8,9,12], or tea extracts, among others, into the reaction solution. This indicates that bio-surfactant sulfonated lignin reaction solutions could reduce interfacial tension, decrease the mass transfer resistance of methane from the gas phase to the liquid phase [8,13], enhance the solubility of methane in the liquid phase, effectively increase methane storage [9], and provide sufficient guest molecules for CH_4_ hydrate nucleation. Lignosulfonates, green bio-surfactants derived from by-products of the paper industry, have demonstrated excellent application potential for hydrate-based energy storage and methane separation from gas mixtures. Ge et al. [9] found that the SL (500 ppm) + H_2_O system exhibited higher methane storage capacity. Moreover, the resulting methane hydrate demonstrated a strong self-preservation effect, enabling stable storage under an atmospheric pressure of 0.1 MPa and 268.15 K (−5 °C), thus significantly reducing the energy required for refrigeration and pressurization in the storage and transportation of solid natural gas (SNG). Wang [14] proposed that the methane storage capacity can reach up to 167–170 *v*/*v* under a 0.5 wt% CL aqueous solution system, thereby reducing induction time to 6 min. Wang L et al. [15] demonstrated that a multi-dimensional synergistic system comprising 1,3-DIOX + SL + spray increased the methane concentration from 30 mol% to 41 mol%. However, studies on methane recovery from LCM via hydrate formation mainly focus on static experimental conditions, and further investigation is needed into system cycle stability, continuous-flow conditions, and the energy requirements for industrial-scale implementation. Recent advances in online methane-monitoring technologies [16,17] have greatly promoted the application of gas separation technology in the industrial field. Furthermore, the incorporation of thermodynamic additives such as tetra-n-butylammonium bromide (TBAB) [10], tetrahydrofuran (THF) [18], and CP [11,19] can significantly improve hydration reaction conditions, enhance the hydration reaction rate, and reduce the energy consumption required for the reaction.

The LCM simulation gas used in this work consists of 30 mol% of CH_4_, 60 mol% of N_2_, and 10 mol% of He, which is different from the simulation gas typically used in previous studies, i.e., comprising 30 mol% CH_4_, 60 mol% N_2_, and 10 mol% O_2_ [11]. This change is due to laboratory safety requirements, as mixing O_2_ and CH_4_ is prone to explosion. Both O_2_ and He in the simulated gas were used as equilibrium gases to represent other gaseous components and impurities present in LCM while minimizing experimental interference. Neither gas participated in the hydration reaction throughout the entire process under normal temperature and low-pressure conditions.

The purpose of this work is to experimentally investigate methane separation from LCM-simulated gas (30 mol% CH_4_, 60 mol% N_2_, and 10 mol% He) using a self-developed experimental apparatus, which can be used for both a pressure stabilization (PS) and a pressure variation (PV) system in the presence of different types of lignosulfonates (SL and CL). Specifically, this work will explore the kinetic reaction characteristics of methane hydrates in a PS system and a PV system under the reaction solutions for lignosulfonates (SL or CL) + CP + H_2_O, combining relevant experimental research results in the presence of lignosulfonates [8,9,14,15].

## 2. Materials and Methods

### 2.1. Materials

The gas used to simulate the LCM gas mixture with a mole composition of 30% CH_4_, 60% N_2_, and 10% He was purchased from Zhengzhou, Yuanzheng Gas company in Henan Province, China, with a reported uncertainty of ±0.05%. Cyclopentane (CP, GC, ≥98%, CAS: 287-92-3, Lot#: C17407861) was purchased from Macklin Reagents company in Shanghai, China. Sodium lignosulfonate (Catalog No. RH697388, Rhawn Reagent, Shanghai, China; CAS: 8061-51-6) and calcium lignosulfonate (Catalog No. RH708773, Rhawn Reagent, Shanghai, China; CAS: 8061-52-7) were obtained with a purity of 96%. Deionized water was used in all experimental runs.

### 2.2. Apparatus and Procedure for Hydrate Reaction

**Figure 1:**
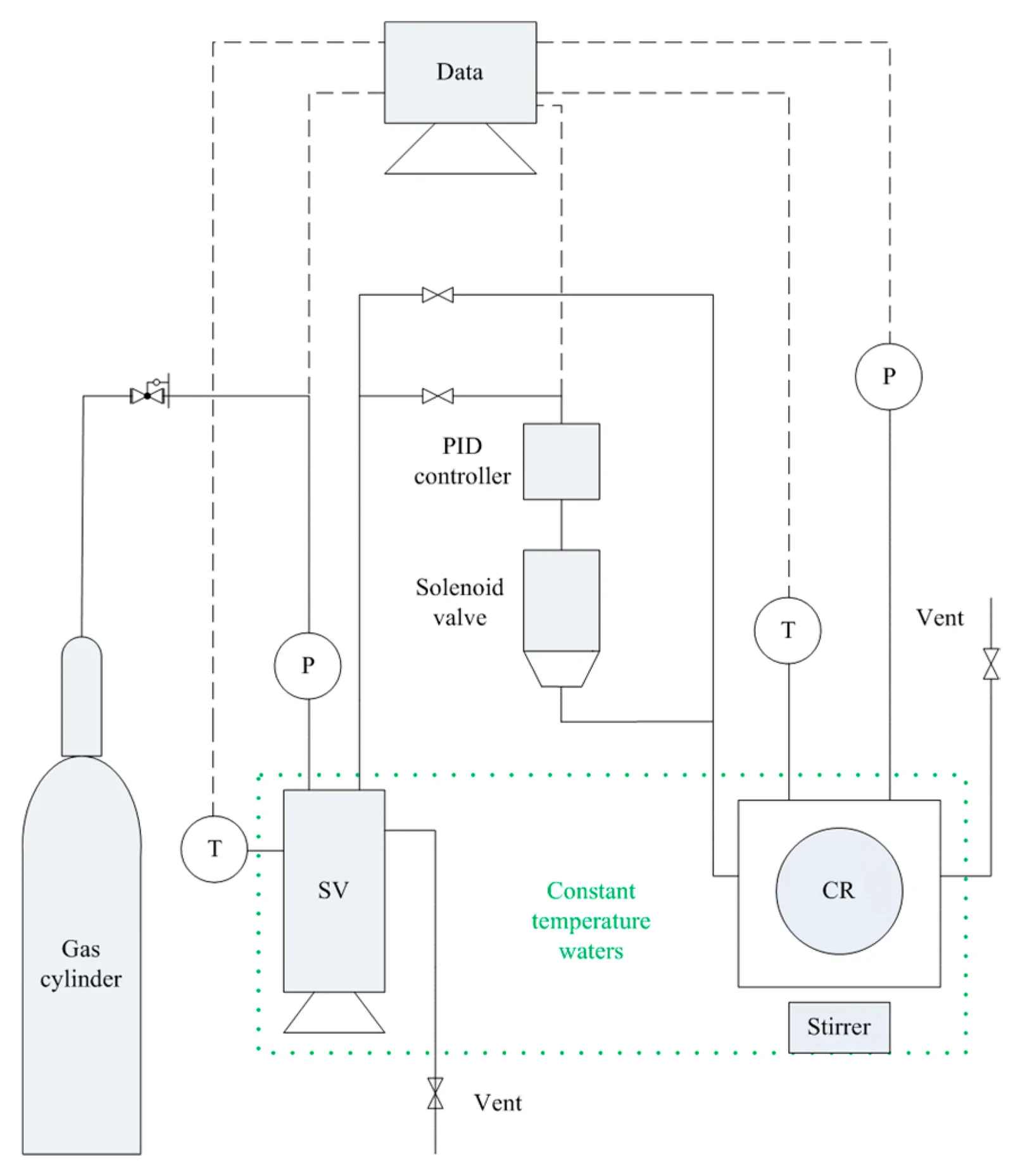
Hydration reaction apparatus of the PS and PV systems.

The self-designed experimental apparatus, designed and manufactured by Haian Hongmai Machinery Co., Ltd, Haian, China, implemented herein for both PS and PV systems is shown in Figure 1. The apparatus was developed by incorporating several design modifications into the conventional PV system, wherein the inlet valves of a solenoid valve (SV) and PID controller will not operate. Detailed descriptions of the PV experimental apparatus and procedures are provided elsewhere in the literature [10,19].

To enable operation under pressure stabilization (PS) conditions, an air compressor, a PID controller, and a solenoid valve were incorporated into the experimental setup. Through a programmed software, fresh LCM gas was automatically transferred from the stability vessel (SV, 800 mL) to the reaction vessel (RV, 250 mL) when the pressure difference between SV and RV exceeded 0.01 MPa, thereby maintaining stable pressure conditions throughout the hydration reaction process. Thus, the gas phase within CR had a higher concentration of CH_4_ gas, which allowed for achieving enhanced efficiency in the hydration reaction rate. The thermostatic waters system is able to achieve a temperature range of 263.15 K to 323.15 K when an SV and RV are placed inside.

The adsorption and hydration reaction solution consisted of deionized water as well as thermodynamic and kinetic additives, such as lignosulfonates, placed inside a high-pressure reactor vessel (RV). The volume of water was fixed at 100 mL, the mass concentration of SL and CL to H_2_O was 500 ppm, the volume ratio of deionized water to cyclopentane was fixed at 10:1 (10 mL), and the cyclopentane mass concentration was 7.0 wt%. The solution comprising lignosulfonate and deionized water was placed in an ultrasonic disperser to ensure uniform dispersion before adding an appropriate amount of CP. The stirring system was fixed at the lower part of the RV, with a stirring speed of 150 r/min. The pressure transmitter was purchased from Chongqing Chuanyi Automation Co., Ltd in Chongqing of China, with an accuracy of ±0.25% F.S (0~60 MPa). The composition of the gas mixtures in the vessels (SV and RV) was measured using gas chromatography (GC) at the end of the hydrate formation experiments.

Prior to the experiments, the RV was cleaned with deionized water and dried, and then filled with 140 mL of the reaction solution. The SV, RV, and tubing were purged three times with the LCM gas mixture to flush out the air remaining in the reactor.

The LCM gas in the gas cylinder entered the SV and RV through valves and tubing. Once the temperature and pressure in the SV and RV had reached their desired values, both vessels were isolated from the gas cylinder by closing the inlet and outlet valves. The stirrer was then started at a fixed speed of 150 rpm, which was defined as the start of the experiments. Temperature and pressure data were continuously recorded by the data acquisition unit every 10 s. The experiment was considered to have stopped when the pressure and temperature remained constant. At the end of the experiment, the composition of the gas remaining in the gas phase of the SV and RV, as well as the gas released from hydrates of the RV, was analyzed via gas chromatography (GC). Three independent experiments were conducted for each set of conditions.

### 2.3. Calculation of Gas Consumption

The calculation of the relevant parameters (*R*, *S*, *r*_30_, etc.) under the PV system have been previously reported by the research group of ref. [19]. The calculation of the LCM gas incorporated into the hydrate crystals under the PS system is divided into two parts: the gas uptake in the SV (*n*_S_) and in the RV (*n*_R_). Both values can be calculated using Equation (1) below.

(1) $$\Delta n_{H}=n_{g,0}-n_{g,t}={\left(\frac{\mathrm{pV}}{\mathrm{ZRT}}\right)}_{0}-{\left(\frac{\mathrm{pV}}{\mathrm{ZRT}}\right)}_{t}$$

where *n*_g,0_ and *n*_g,t_ represent the molar quantity of gas in the reactor at time 0 and at time t, respectively; *P* is the pressure of the container; *V* is the volume of gas phase; *T* is the temperature of the gas phase; R represents the ideal gas constant; and *Z* is the compressibility factor calculated using the Pitzer correlation [20]. It should be noted that the calculation of gas uptake under the PS system should be the sum of both SV and RV, and the gas uptake of the PV system can be calculated using the formula found in reference [9].

The hydrate average formation rate of this work is typically regarded as the gas uptake and calculated via the forward difference method [20] as follows:

(2) $$r={\left(\frac{d\Delta n_{H}}{\mathrm{dt}}\right)}_{t}=\frac{\Delta n_{H,t+\Delta t}-\Delta n_{H,t}}{\Delta t},\Delta t=10s$$

The average of these rates is calculated every 30 min and reported.

(3) $$r_{30\min}=\frac{{\left(\frac{d\Delta n_{H}}{\mathrm{dt}}\right)}_{1}+{\left(\frac{d\Delta n_{H}}{\mathrm{dt}}\right)}_{2}+\ldots +{\left(\frac{d\Delta n_{H}}{\mathrm{dt}}\right)}_{m}}{m},m=180$$

### 2.4. Calculation of CH_4_ Recovery Rate

Methane recovery (*R*) and the separation factor (*S*) are commonly used to evaluate the efficiency of gas mixture separation via hydrate formation and have been adopted by Linga et al. [21,22]. In this study, methane recovery was calculated from a methane mole balance based on the experimental PVT data.

In this work, the moles of CH_4_ in the hydrate phase within RV was calculated as follows:

(4) $$\Delta n_{{\mathrm{CH}}_{4}}^{H}=n_{{\mathrm{CH}}_{4}}^{0}-n_{{\mathrm{CH}}_{4}}^{g}=(n_{{\mathrm{CH}}_{4}}^{S,0}+n_{{\mathrm{CH}}_{4}}^{R,0})-(n_{{\mathrm{CH}}_{4}}^{S,g}+n_{{\mathrm{CH}}_{4}}^{R,g})$$

where $\Delta n_{{\mathrm{CH}}_{4}}^{H}$ is the moles of CH_4_ in the hydrate phase within RV, $n_{{\mathrm{CH}}_{4}}^{0}$ is the sum of the moles of CH_4_ in the gas phase of SV ($n_{{\mathrm{CH}}_{4}}^{S,0}$) and RV ($n_{{\mathrm{CH}}_{4}}^{R,0}$) before the reaction begins, and $n_{{\mathrm{CH}}_{4}}^{g}$ is the sum of the moles of CH_4_ in the gas-phase spaces of SV ($n_{{\mathrm{CH}}_{4}}^{S,g}$) and RV ($n_{{\mathrm{CH}}_{4}}^{R,g}$) at the end of the reaction.

The CH_4_ recovery (*R*) refers to the efficiency of recovering CH_4_ from the LCM gas, and the separation factor (*S*) indicates the extent of separating CH_4_ from the LCM gas. The CH_4_ recovery (*R*) was calculated as follows:

(5) $$R=\frac{\Delta n_{{\mathrm{CH}}_{4}}^{H}}{n_{{\mathrm{CH}}_{4}}^{\mathrm{feed}}}=\frac{\left(n_{{\mathrm{CH}}_{4}}^{S,0}+n_{{\mathrm{CH}}_{4}}^{R,0})-(n_{{\mathrm{CH}}_{4}}^{S,g}+n_{{\mathrm{CH}}_{4}}^{R,g}\right)}{(n_{{\mathrm{CH}}_{4}}^{S,0}-n_{{\mathrm{CH}}_{4}}^{S,g})+n_{{\mathrm{CH}}_{4}}^{R,0}}$$

where $n_{{\mathrm{CH}}_{4}}^{\mathrm{feed}}$ and $\Delta n_{{\mathrm{CH}}_{4}}^{H}$, respectively, explain the methane gas uptake during the whole hydration reaction process and the methane gas uptake by the hydrate phase.

The calculation of the separation factor (*S*) in this work was defined using the following equation:

(6) $$S=\frac{n_{{\mathrm{CH}}_{4}}^{H}(n_{N_{2}}^{\mathrm{gas}}+n_{\mathrm{He}}^{\mathrm{gas}})}{n_{{\mathrm{CH}}_{4}}^{\mathrm{gas}}(n_{N_{2}}^{H}+n_{\mathrm{He}}^{H})}$$

where $n_{N_{2}}^{\mathrm{gas}}$, $n_{\mathrm{He}}^{\mathrm{gas}}$, and $n_{{\mathrm{CH}}_{4}}^{\mathrm{gas}}$ are the moles of CH_4_, N_2_, and He in the gas phase of RV at the end of the experiments, respectively. $n_{{\mathrm{CH}}_{4}}^{H}$, $n_{N_{2}}^{H}$, and $n_{\mathrm{He}}^{H}$ are the moles of CH_4_, N_2_, and He in the hydrate phase of RV at the end of the experiments, respectively.

## 3. Results

In this study, a combined system integrating a PS system (which enables the continuous supply of LCM gas from SV to RV to maintain high reactor pressure), thermodynamic relaxation (CP), and dynamic enhancement (lignin) was developed. Based on our previous studies, we found that sodium lignosulfonate reaches its optimal gas storage capacity at a mass concentration of 500 ppm, and a higher methane recovery rate from LCM was confirmed to occur at a mass percent of 7 wt% for CP. In this work, we explore the kinetic characteristics of CH_4_ recovery from LCM gas via hydrate formation under PS and PV systems with SL and CL as dynamic additives, and evaluate the influence of thermodynamic promoters on CP. The experimental conditions were set as follows: a temperature of 275.15 K, a pressure of 3.0 MPa, a stirring speed of 150 r/min, and a CP mass percent of 7 wt%. The lignins, sodium lignosulfonate and calcium lignosulfonate, with a mass concentration of 500 ppm, were incorporated as a dynamic additive in the experimental process. Table 1 displays the experimental conditions. Each experiment was repeated three times. The effects of hydrate formation on the PS and PV systems were investigated through experiments PV 1–3 and PS 1–3, whereas the hydrate growth characteristics of coal mine methane under different lignosulfonates were examined in experiments PS 1–6.

The induction time (*t_ind_*) is defined as the time at which hydrate nucleation occurs and can be determined by visual observations [23], pressure variations [24], or a sudden increase in the temperatures of the gas and hydrate phases [25]. Generally, *t*_ind_ decreased with increasing driving force, while the induction times were relatively close under three conditions: *t*_ind_ = 10.3 min, 18.3 min, and 11 min, in the PV-SL, PS-SL, and PS-CL systems, respectively. This is most likely due to the high nucleation rate of methane hydrates under the three experimental conditions. The gas uptake (Δ*n*_H_), CH_4_ recovery (*R*), and separation factor (*S*) were calculated using Equations 1, 4, 5, and 6, respectively. The combined standard uncertainties in Δ*n*_H_ were also calculated from the uncertainties in *P*, *T*, *V*, and *Z*. The *t*_90_ values, i.e., the time required for the gas uptake to reach 90% [15], were 156.7 min, 168.3 min, and 347.3 min, corresponding to the PV-SL, PS-SL, and PS-CL systems, respectively. These values indicate that the PS system continuously provides fresh gas to the RV compared to the PV system, thus increasing the hydration reaction time; this can be seen from the *t*_90_ values obtained from experiments PV 1–3 and PS 1–3. On the other hand, a comparison of the *t*_90_ values from experiments PS 1–6 showed that the hydration reaction time of the CP + CL system was longer than that of the CP + SL system, which indicates that the CP+SL system may offer advantages in terms of energy efficiency for CH_4_ recovery from LCM gas via hydrate formation. The separation factor (*S*) calculated herein is suitable for comparing separation performances under the same system (whether PV or PS). However, direct comparisons of S between PS and PV systems can lead to systematic inaccuracies because fresh LCM gas is continuously injected into the RV from the SV, while the PV system is a closed batch system without continuous gas feeding. These differences make cross-mode comparisons of *S* unreliable.

**Figure 2:**
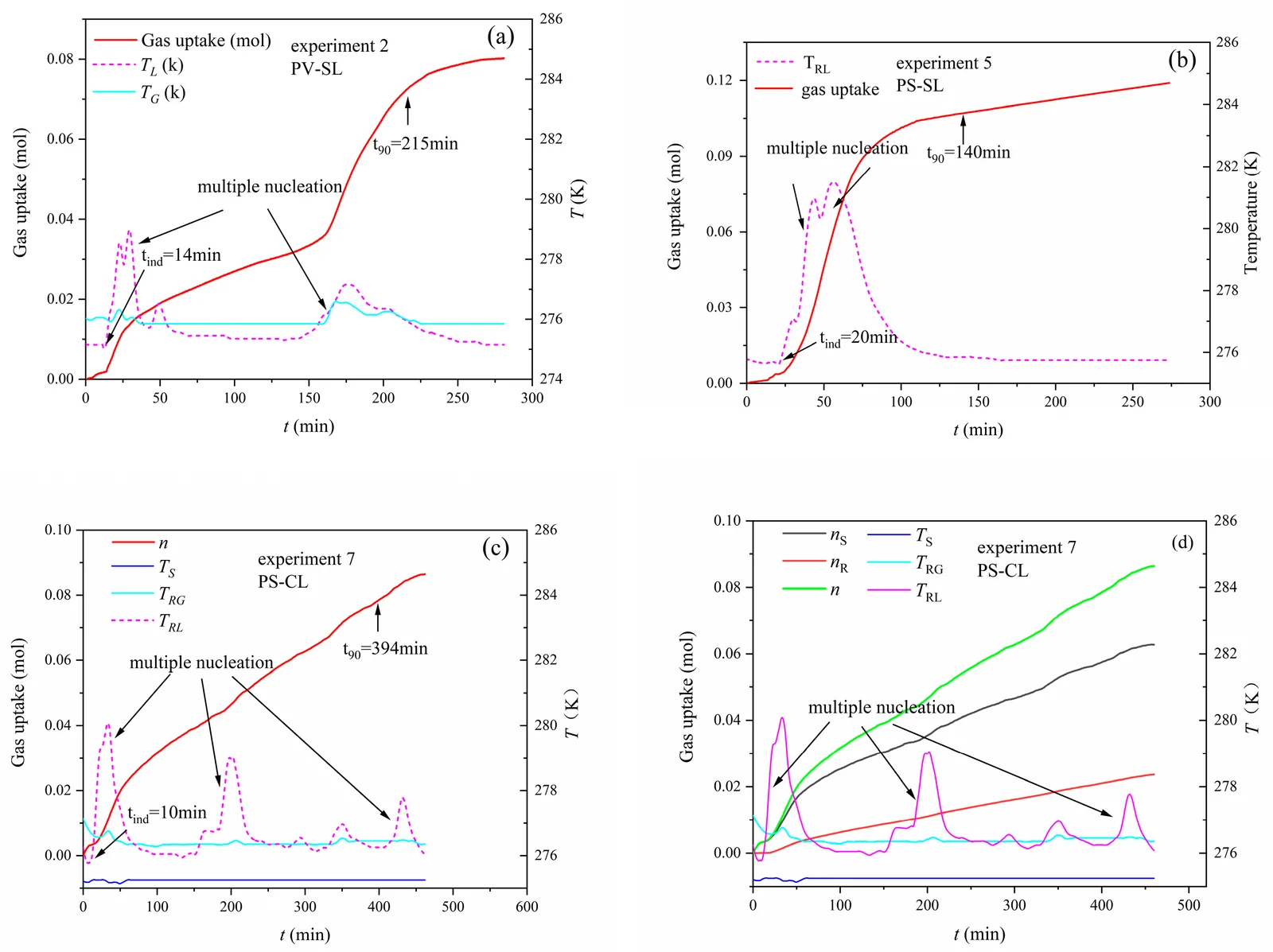
Temperature-variation-and-gas-uptake curve for the experiment carried out at 275.15 K, 3.0 MPa, and CP (7.0 wt %) in the presence of different lignin under the PV and PS systems. (**a**) Temperature variation and gas uptake curve for PV-SL system, experiment PV-2; (**b**) Temperature variation and gas uptake curve for PV-SL system, experiment PS-2; (**c**) Temperature variation and gas uptake curve for PV-SL system, experiment PS-4; (**d**) Gas uptake variation status of SV, RV and SV + RV for PV-SL, experiment PS-4.

Figure 2 shows the temperature-variation-and-gas-uptake curve for the experiment carried out at 275.15 K and 3.0 MPa in the presence of different lignins and CP (7.0 wt %) under both a PV and PS system, with the stirring speed fixed at 150 r/min. The induction time was determined using a temperature curve, pressure drop curve, and visual observations. The figure shows that the overall induction time is relatively short (10~20 min), indicating that the first large-scale nucleation of hydrates occurred shortly after the onset of the experiment. Figure 2a,b shows the temperature-variation-and-gas-uptake curve under different reaction systems (PV and PS). It can be seen in Figure 2b that the maximum temperature of the liquid phase for RV (T_max_ = 281.5 K) was significantly higher than that (T_max_ = 278.9 K) in Figure 2a. This temperature difference suggests that hydrate formation was more extensive under the PS system compared to the PV system. At the same time, the gas uptake curve under the PS system indicates that the multiple nucleation of methane hydrates primarily occurred from induction time to 100 min. Hydrate formation had largely progressed by 100 min, although 90% of the final gas uptake was not reached until 140 min (*t*_90_ = 140 min). In contrast, collective multiple nucleation phenomena appeared twice under the PV system, thus resulting in a longer hydrate reaction process (*t*_90_ = 215 min), as indicated by the gas uptake curve. Meanwhile, the PS + SL system exhibited a higher gas uptake of 0.1142 mol. Further analysis of the collective multiple nucleation phenomena can be carried out using microscopic imaging and Raman spectroscopy. 

The temperature-variation-and-gas-uptake curve under different lignins (SL and CL) is provided in Figure 2b,c. The highest temperature during hydrate formation under the PS + SL system (T_max_ = 281.5 K) was higher than that of the PS + CL system (T_max_ = 280.1 K), which confirmed that more methane hydrates were generated in a shorter period of time under the PS + SL system. In addition, the *t*_90_ of the CL system was 347.3 min, much higher than that of the SL system. This corresponds with the temperature variation, resulting in a longer hydration reaction time. Furthermore, the CL system exhibited lower gas uptake than the SL system. Overall, a faster hydration reaction rate and a higher gas uptake were achieved under the PS + SL system compared with the PV + SL and PS + CL systems. This is due to the combined action of adsorption (lignin) and hydration (CP).

Figure 2d shows the gas uptake variation status of SV, RV, and SV + RV for experiment 5, carried out at 275.15 K and 3.0 MPa in the presence of CL and CP, under the PS system.

**Figure 3:**
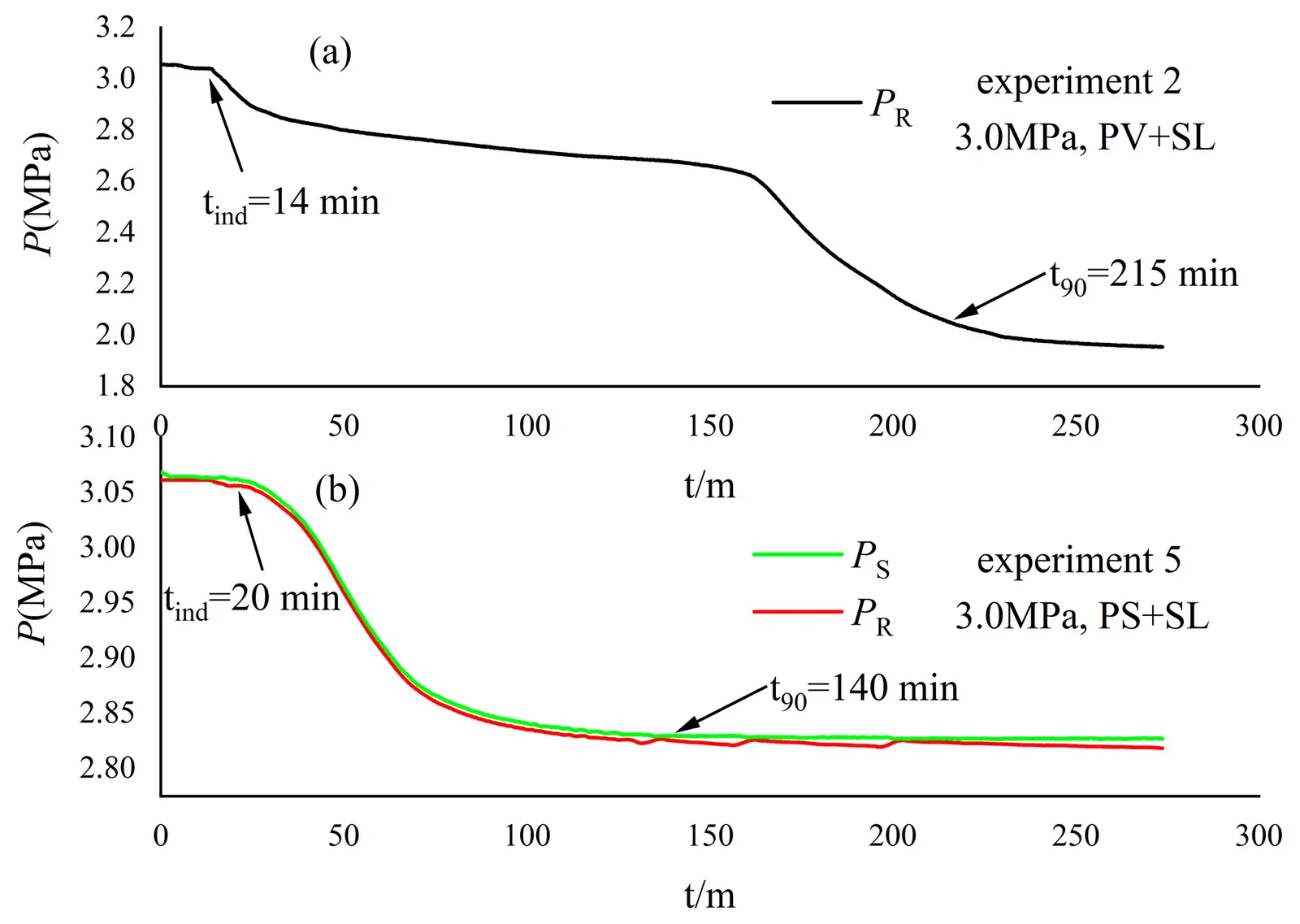
Pressure variation curve for the experiment carried out at 275.15 K, 3.0 MPa, and CP (7.0 wt %) under PV and PS systems. (**a**) Pressure variation curve for PV+SL system, experiment PV-2; (**b**) Pressure variation curve for PS+SL system, experiment PS-2.

Figure 3 shows the pressure variation curve for the experiment carried out at 275.15 K, 3.0 MPa and CP (7.0 wt %) under both PV and PS systems. It can be seen from Figure 3a,b that the pressure drop curve is relatively consistent with the gas consumption curve in Figure 2a,b. Furthermore, the induction time (*t*_ind_) determined by the pressure variation curve in Figure 3 agrees well with that identified in Figure 2. The extensive nucleation of hydrates was also inevitably accompanied by a sharp decrease in pressure.

**Figure 4:**
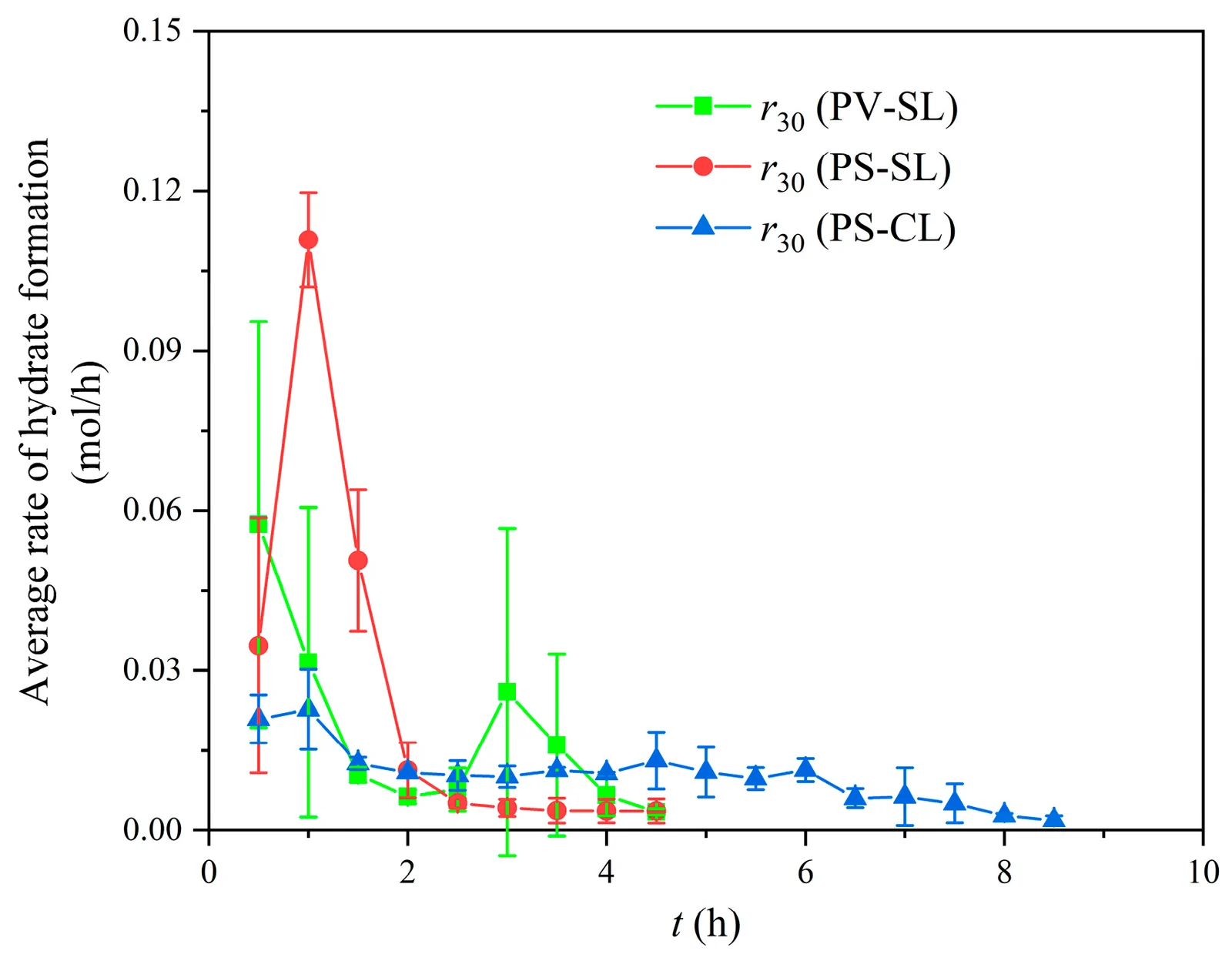
Effect of different lignins on the average rate of hydrate formation from LCM gas under PS and PV systems (time zero is the nucleation point). Error bars represent standard deviation of three replicate measurements.

Figure 4 compares the average rate of hydrate formation from LCM gas in the presence of different lignins (SL and CL) under the PS and PV systems; time zero is the nucleation point. The rate of hydrate formation and its 30 min average (*r*_30_) were calculated using Equations (2) and (3). The PS + SL system exhibited a higher average hydrate formation rate during the first 100 min, with the highest *r*_30_ reaching 0.1108 mol/h at 60 min. This may be due to the continuous supply of fresh LCM gas from the stabilization vessel (SV) into the reaction vessel (RV), thus resulting in a higher concentration of CH_4_ in the gas phase of the RV. In contrast, the average rate of hydrate formation under the PV + SL system gradually decreased after reaching a maximum of 0.0574 mol/h at 30 min. This decline may be attributed to the continuous consumption of methane during extensive hydrate nucleation and growth, which progressively reduced the methane concentration in the gas phase of the RV. This trend was consistent with the gas uptake and temperature variations shown in Figure 2a,b. Furthermore, the 30-minute average hydrate formation rate exhibited relatively small fluctuations, indicating that the entire hydration reaction process proceeded gradually. The *r*_30_ remained high in the first 60 min (the highest *r*_30_ was 0.0227 mol/h) and then maintained at 0.010 mol/h. This trend is consistent with that of the gas uptake and temperature variation shown in Figure 2c. It also proves that a longer hydration reaction time and more energy would be required under the PS + CL system compared with the PS + SL system.

**Figure 5:**
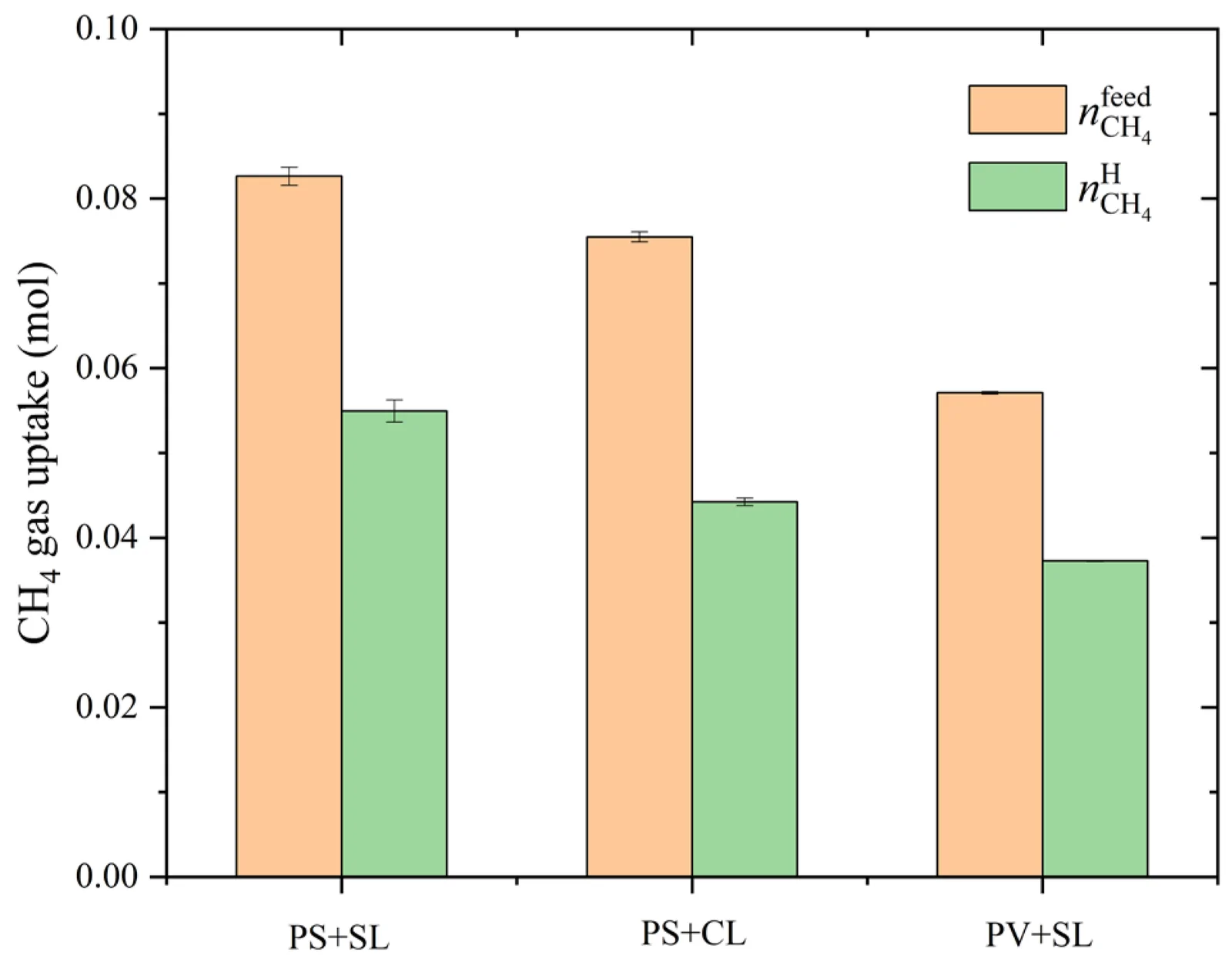
Comparison chart of the average CH_4_ gas uptake and feed in the presence of different lignins under PS and PV systems. Error bars represent standard deviation of three replicate measurements.

Figure 5 shows the average CH_4_ gas uptake and feed in the presence of different lignins under the PS and PV systems. $n_{{\mathrm{CH}}_{4}}^{\mathrm{feed}}$ values were 0.0826 mol, 0.0755, mol and 0.0571 mol under the PS + SL, PS + CL, and PV + SL systems, respectively. $\Delta n_{{\mathrm{CH}}_{4}}^{H}$ values were 0.0550 mol, 0.0442 mol, and 0.0373 mol under the PS + SL, PS + CL, and PV + SL systems, respectively. $n_{{\mathrm{CH}}_{4}}^{\mathrm{feed}}$ and $\Delta n_{{\mathrm{CH}}_{4}}^{H}$ values were both the highest under the PS + SL system.

**Figure 6:**
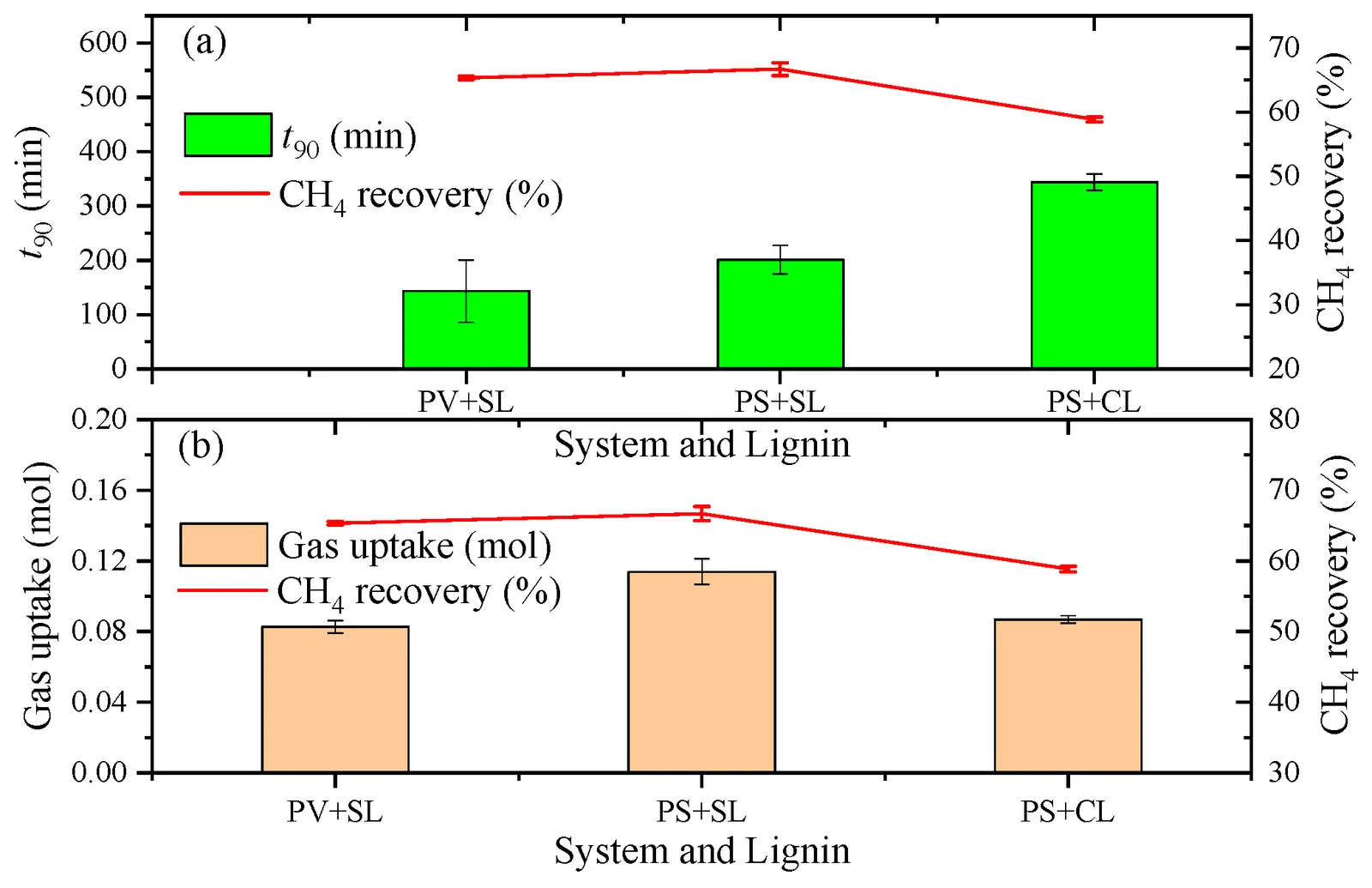
The average CH_4_ recovery, gas uptake, and *t*_90_ for methane separation from LCM gas via hydrate formation with different lignins under PS and PV systems. Error bars represent standard deviation of three replicate measurements. (**a**) The average CH_4_ recovery and *t*_90_ in different lignosulfonates under PS or PV system; (**b**) The average CH_4_ recovery and gas uptake in different lignosulfonates under PS or PV system.

Figure 6 presents the average CH_4_ recovery, gas uptake, and *t*_90_ for methane separation from LCM gas via hydrate formation using different lignins under the PS and PV systems. In this work, the highest methane recovery (R = 66.5%) and gas uptake (0.1142 mol) were observed under the PS+SL system. This result may be attributed to two factors: First, the continuous supply of fresh CM gas into the RV under the PS system maintained a higher CH_4_ concentration in the gas phase than under the PV system. Second, the SL system exhibited better methane storage capacity and recovery efficiency compared with the CL system. As shown in Figure 6, a more efficient system (i.e., the PS + SL + CP system) was developed for methane enrichment from LCM, achieving CH_4_ recovery and gas uptake through the combined action of absorption and hydration.

**Figure 7:**
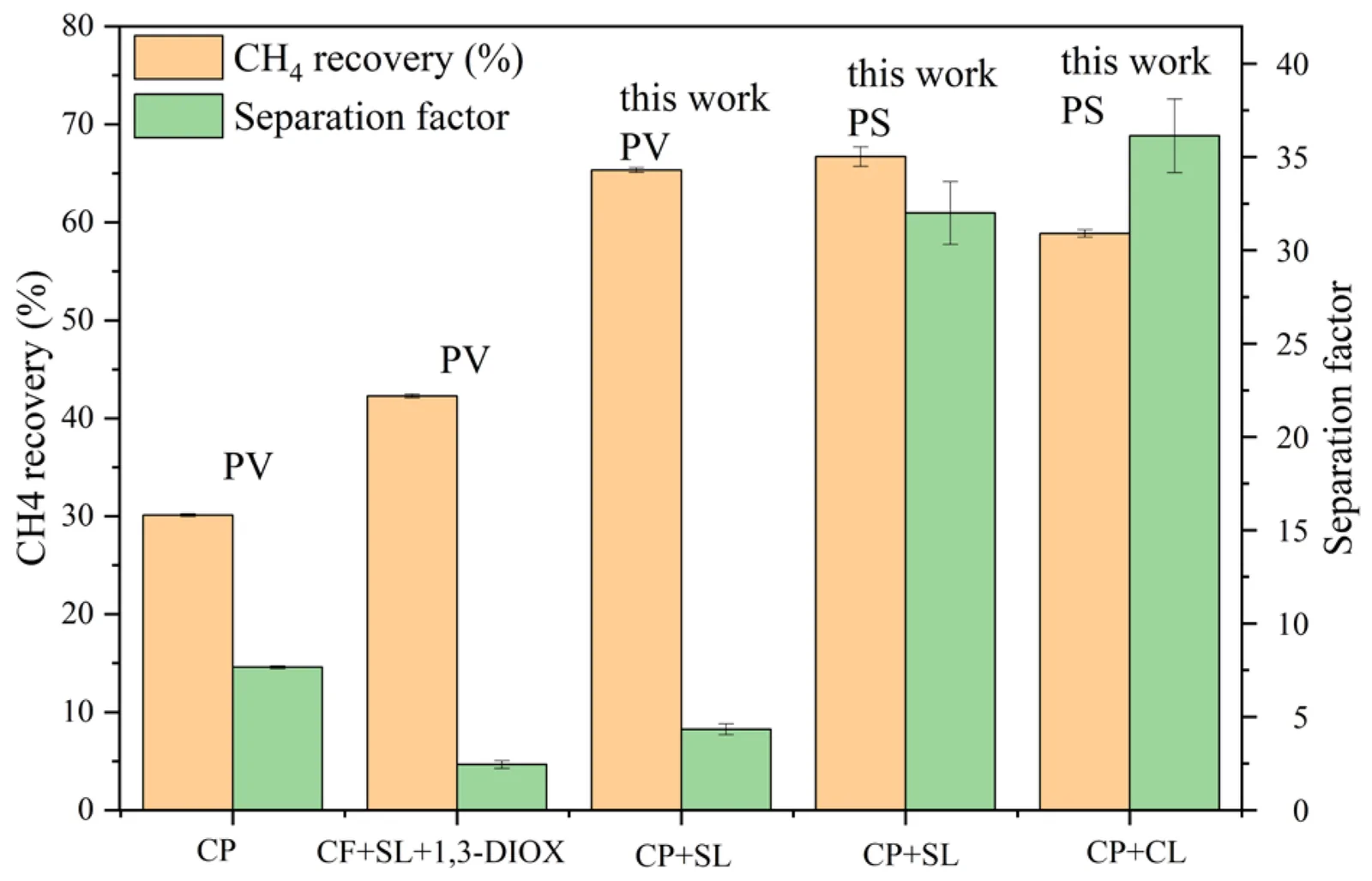
The average CH_4_ recovery and separation factor obtained through methane separation from LCM gas via hydrate formation in different systems (CP [11], CF+SL+1,3-DIOX [15]). Error bars represent standard deviation of three replicate measurements.

At present, there are numerous experimental studies on methane separation via hydrate formation using CP as a thermodynamic additive [26,27], but research on SL [28] as a kinetic additive to separate methane from low-concentration coalbed via hydrate formation remains scarce. Figure 7 presents selected research results on methane separation from LCM gas using CP and SL as additives. In the present work, CH_4_ separation from LCM gas was conducted under the experimental conditions of 275.15 K, 3.0 MPa, 7 wt% CP, 500 ppm SL, and PS systems, and the average CH_4_ recovery (*R*) obtained was 66.5%. This value of CH4 recovery increased by 55% and 37%, respectively, as compared to those obtained in the presence of CP (30%) [11] and CF (copper foam) + 1,3-DIOX (1,3-dioxolane) + SL (42.15%) [28]. Figure 7 also shows the separation factor (*S*) under the PV system, as the corresponding value for the PS system may contain some inaccuracies (for detailed data, refer to Table 1).

## 4. Conclusions

In this work, an integrated system combining pressure stabilization, a surfactant, a thermodynamic accelerator, and hydration was developed for methane separation from LCM gas via hydrate formation, increasing the CH_4_ concentration from 30% to 69%. This study focused on the characteristics of methane separation from CM gas via hydrate crystallization with the incorporation of different lignins (SL and CL) and under different reaction systems (PS and PV systems). We used the following fixed experimental conditions: 275.15 K, 3.0 MPa, and 7 wt% CP. The maximum methane recovery and gas uptake values were confirmed as 66.5% and 0.1142 mol under three conditions (PV + SL, PS + SL, PS + CL). Therefore, the combined system of pressure stabilization, surfactant, thermodynamic accelerator, and hydration developed in this work can be used as an effective format for CH_4_ separation from LCM gas via hydrate formation, providing a feasible technical route for hydrate separation processes.

## Figures and Tables

**Table 1:** Experimental conditions and results for CH_4_ recovery from low-concentration coalbed methane via hydrate formation with the coupling effect of CP and lignins under PV and PS systems ^a^.

Exp. ^b^	Lignin (ppm)	*t_i_*_nd_ (min)	*t*_90_^c^ (min)	Δ*n*_H_ (mol)	*u*(Δ*n*_H_) (mol)	${x_{{\mathrm{CH}}_{4}}^{\mathrm{gas}}}^{d}$ (%)	${x_{{\mathrm{CH}}_{4}}^{H}}^{e}$ (%)	*R* (%)	*S*
PV-1	500(SL)	8	103	0.0851	0.0057	19.0	69.6	65.5	4.0
PV-2		14	215	0.0802	0.0053	18.1	69.5	65.2	4.5
PV-3		9	152	0.0824	0.0054	18.2	69.5	65.1	4.5
PS-1	500(SL)	13	173	0.1088	0.0072	20.9	59.5	66.0	33.9
PS-2		20	140	0.1190	0.0079	17.0	60.7	67.4	30.7
PS-3		22	192	0.1149	0.0077	17.0	59.2	66.1	31.4
PS-4	500(CL)	10	394	0.0864	0.0059	19.0	54.8	59.1	38.3
PS-5		8	333	0.0853	0.0057	19.0	53.4	58.6	35.7
PS-6		15	315	0.0835	0.0058	19.2	56.1	58.1	34.4

a Standard uncertainties u are u(*T*) = 0.1 K and u(*P*) = 6 kPa. b PV is pressure variation system experiments and PS is pressure stabilization system experiments. c The time required for the gas uptake to reach 90%. d Methane concentration in the gas phase ($x_{{\mathrm{CH}}_{4}}^{\mathrm{gas}}$) measured at the end of the experiment by GC. e Methane concentration in the hydrate phase ($x_{{\mathrm{CH}}_{4}}^{H}$) measured at the end of hydrate dissociation by GC.

## Data Availability

The original contributions presented in this study are included in the article. Further inquiries can be directed to the corresponding author.

## Abbreviations

The following abbreviations are used in this manuscript:

CP	Cyclopentane
SL	Sodium lignosulfonate
CM	Coalbed methane
PS	Pressure stability
PV	Pressure variation
SV	Stabilization vessel
RV	Reaction vessel
GC	Gas chromatograph
S	Separation factor
R	CH_4_ recovery or split fraction
